# Correlation between vascular endothelial growth factor, soluble urokinase plasminogen activator receptor, and tricuspid annular plane systolic excursion/systolic pulmonary artery pressure ratio in group E chronic obstructive lung disease

**DOI:** 10.1590/1806-9282.20240589

**Published:** 2024-11-11

**Authors:** Sami Deniz, Burcu Uludağ, Ferhat Demirci

**Affiliations:** 1 University of Health Sciences Turkey, İzmir Faculty of Medicine – İzmir, Turkey.; 2 Dr. Suat Seren Chest Diseases and Thoracic Surgery Research and Education Hospital – İzmir, Turkey.

**Keywords:** VEGF-A, Pulmonary hypertension, COPD

## Abstract

**OBJECTIVE:**

Vascular endothelial growth factor is a signaling protein created by cells performing important bodily functions. Vascular endothelial growth factor is abundant in the lung, and plasma levels are elevated in patients with severe pulmonary arterial hypertension. An association between soluble urokinase plasminogen activator receptor, an inflammatory biomarker, and soluble urokinase plasminogen activator receptor levels and interstitial pulmonary and vascular involvement (e.g., development of pulmonary hypertension) has been shown in SSc patients. The tricuspid annular plane systolic excursion/systolic pulmonary artery pressure ratio, which has been recommended as a useful diagnostic tool in the last guideline, is one of the additional echocardiographic signs suggestive of pulmonary hypertension. We aimed to examine whether these biomarkers contribute to the diagnosis and management of pulmonary hypertension.

**METHODS:**

Patients with group E chronic obstructive lung disease were included in this prospective study. Demographic data, echocardiographic signs about the right ventricle (right atrium area, tricuspid annular plane systolic excursion/systolic pulmonary artery pressure, fractional area change, and right ventricular outflow tract), and peripheral blood analysis were examined and recorded.

**RESULTS:**

A total of 70 patients, 12 of whom were female, were analyzed in the study. The mean age was 66.6±8.7 years. The mean vascular endothelial growth factor-A and soluble urokinase plasminogen activator receptor were 91.05±70.7 and 955.8±571.1, and their Pearson correlation coefficients between vascular endothelial growth factor-A and tricuspid annular plane systolic excursion/systolic pulmonary artery pressure, and soluble urokinase plasminogen activator receptor and tricuspid annular plane systolic excursion/systolic pulmonary artery pressure ratio were 0.341 (p=0.004) and −0.045 (p=0.70), respectively. The linear regression model included four variables with significant correlation (vascular endothelial growth factor-A, right atrium area, fractional area change, and right ventricular outflow tract). Three steps were performed, and adjusted r^2^ was 0.22, 0.22, 0.20, and p<0.001 for each step. Vascular endothelial growth factor-A and right ventricular outflow tract remained in the last step. It was detected a standardized coefficient beta of 0.322 (p=0.004) and a 95%CI 0.000–0.001 for vascular endothelial growth factor-A.

**CONCLUSION:**

Vascular endothelial growth factor-A is correlated with the tricuspid annular plane systolic excursion/systolic pulmonary artery pressure ratio and not with soluble urokinase plasminogen activator receptor.

## INTRODUCTION

Pulmonary hypertension (PH) is defined by mean pulmonary arterial pressure (mPAP) >20 mmHg via right heart catheterization (RHC) at rest. The clinical classification of PH is categorized into five groups based on clinical conditions and presentations. Among these groups, the second most common cause of PH is associated with lung diseases and hypoxia^1^. The most common disease in this group is chronic obstructive lung disease (COPD). Although the prevalence of PH in COPD is, in general, dependent on the severity of the disease^2^, severe PH has been detected in 1–5% of advanced disease^3^. In the diagnostic phase, transthoracic echocardiography (TTE) is still the primary non-invasive tool. Right ventricle (RV) function is assessed by a number of measures, including tricuspid annular plane systolic excursion (TAPSE), RV fractional area change (FAC), tissue Doppler peak systolic velocity at the tricuspid annulus, and right ventricular myocardial performance index^4^. Right atrium (RA) area, right ventricle/left ventricle (RV: LV) ratio, left ventricle (LV) eccentricity index, tricuspid regurgitation velocity (TRV), systolic pulmonary artery pressure (sPAP), TAPSE, FAC, RV free-wall strain, and the TAPSE/sPAP ratio may help diagnose PH^1^. The presence of RVOT Doppler notching has been shown to be strongly suggestive of pulmonary hypertension in the setting of elevated PVR^4^. Furthermore, the guideline recommended that the TAPSE/sPAP ratio be used as additional echocardiographic signs for ventricles. If the TAPSE/sPAP ratio is less than 0.55, it is accepted as an additional echocardiographic sign for ventricles^1^.

Vascular endothelial growth factor (VEGF) is a signaling protein created by cells performing important bodily functions. Lung tissue is very rich in VEGF protein; many different lung cells produce VEGF and also respond to VEGF. Air pollutants, heavy smoking, and other toxic gas exposures trigger overexpression of VEGF and fibroblast growth factor-2 (FGF-2) in lung tissue with activation of the inflammatory cascade, which is a major cause of the development of abnormal angiogenesis in the lung^5^. Several studies in animal models have shown increased angiogenesis in lung tissue associated with endothelial cell overproliferation, which is accountable for the development of PH^6^.

The urokinase plasminogen activator (uPA) and its receptor (uPAR) system are ample in various cell types, including vascular endothelial cells. They are key regulators of cross-reactions between vascular inflammation, immunity, and coagulopathy. Soluble uPAR (suPAR) is a cleavage product of the uPA/uPAR system; its levels are thought to reflect the overall activity of the system^7,8^. suPAR, which is an inflammatory biomarker, is elevated in patients with ongoing inflammatory processes and has been detected to have prognostic value in varied types of cancer^9,10^. A study showed that suPAR was one of the predictors of post-capillary PH, and they could discriminate between pre- and post-capillary PH^11^.

In our study, we aimed to investigate the relationship between suPAR, VEGF, and echocardiographic parameters and whether they contribute to the diagnosis and management of PH.

## METHODS

In this prospective study, patients with group E COPD were screened. The Institutional Review Board approved the study for the Human Studies and Ethics Committee. A written informed consent form was obtained from the patients.

We screened 961 patients with at least one hospitalization in the previous year. A total of 70 patients who met the inclusion criteria were included in the study. Vital signs were inspected. Routine blood examinations, troponin T, D-dimer, and pro-brain natriuretic peptide (BNP) were examined. Post-bronchodilator pulmonary function tests (PFT), electrocardiogram, and echocardiography (by an experienced PH cardiologist) were performed in all patients. Demographic data were also recorded. None of the patients used long-term oxygen therapy because they did not have respiratory failure.

### Inclusion criteria

Patients with group E COPD, a definitive diagnosis of COPD (post-bronchodilator PFT; FEV^1^/FVC<70), consistent with clinical compliance and exposures.

### Exclusion criteria

Cancer, pulmonary embolism [it was excluded by Wells score, D-dimer, and thorax CT angio when necessary (thoracic angio-CT scan rate was 71%)], coexistence of asthma, interstitial lung disease (ILD), and other lung diseases, pneumonia, immunological disorders, presence of infection, left heart failure (LHF), congenital heart diseases, left heart valvular diseases, as well as pulmonary stenosis, chronic liver, renal diseases (self-reported, with liver and renal function tests in blood, complete urine test, ultrasound in suspected patients, and abdominal ultrasonography rate 53%), and head trauma in the previous year (self-reported).

### Vascular endothelial growth factor and soluble urokinase plasminogen activator receptor

Blood samples (8.5 mL) were taken on the first day of admission, early in the morning, after overnight fasting, from the antecubital vein, and were processed (centrifugation at 3,000 rpm for 10 min) within 30 min of collection. The serum was aliquoted and stored at −20 °C until analysis. The measurements were double-batched and tested. The concentrations of VEGF were measured using commercial, enzyme-linked immune-sorbent assay technology (Human VEGF-A ELISA Kit, Ray Biotech Inc., 3607 Parkway Lane Suite Peachtree Corners, GA 30092, USA, Catalog Number ELH-VEGF, Lot Number 0304220196). The VEGF detection range was 10–6,000 pg/mL, and the assay's sensitivity was observed at 10 pg/mL. The concentrations of uPAR were measured using a commercial enzyme-linked immune-sorbent assay technology (Human uPAR ELISA Kit, Ray Biotech Inc., 3607 Parkway Lane Suite Peachtree Corners, GA 30092, USA, Catalog Number ELH-uPAR, Lot Number 0304220028). The uPAR detection range was 15–4,000 pg/mL, and the assay's sensitivity was observed at 15 pg/mL.

### Cardiovascular evaluation

Resting TTE was performed by a cardiologist who was blinded to all other data using a Philips Affiniti 50 echocardiography device (Philips Medical System Andover, Andover, USA). Images were obtained from the left parasternal long-axis and short-axis, apical four-chamber, and subcostal views. LV wall thicknesses, LV end-diastolic and end-systolic diameters, and LA dimensions were measured. Left ventricle ejection fraction (LVEF) was calculated using the Teichholz formula. To assess the diastolic functions of the LV, the mitral inflow velocities were evaluated from the apical four-chamber view. The peak velocity of early diastolic transmitral flow (E), peak velocity of atrial systolic transmitral flow (A), E/A ratio, and E wave deceleration time were measured by using pulsed-wave Doppler echocardiography. The early diastolic velocity of the lateral mitral annulus (Em) was recorded with tissue Doppler imaging.

RV basal, mid-level, and longitudinal diameters and RA area were estimated at end-diastole from an RV-focused apical four-chamber view. RV wall thickness was measured in diastole from the subcostal view. RV systolic function was evaluated using FAC, TAPSE, and tissue Doppler-derived tricuspid lateral annular systolic velocity (Sa). Doppler parameters were used to evaluate the diastolic function of the RV. Tricuspid E wave velocity, A wave velocity, deceleration time, E/A ratio, and tissue Doppler-derived diastolic early (Ea) and late (Aa) velocities of the lateral tricuspid annulus were measured^6^.

We also measured TRV using continuous-wave Doppler echocardiography from the apical four-chamber view. sPAP was calculated as follows: 4 × (TRV)²+RAP. RAP was estimated based on the width of the inferior vena cava and its collapse during inhalation.

### Statistical analysis

The data were analyzed in SPPS version 28. The Kolmogorov-Smirnov test was used for the normal distribution tests of numerical variables, which were normally distributed. These variables are presented as the mean±SD. After the Pearson correlation test, the linear regression analysis was performed to estimate the relation between the VEGF, suPAR, and TAPSE/sPAP ratio. The level of statistical significance was determined to be 0.05.

## RESULTS

A total of 70 patients with group E COPD, 12 of whom were female, were included in the study. The mean age was 66.6±8.7 years. A total of 32 patients had hypertension (HT) and/or diabetes mellitus (DM). There was no comorbidity among the patients except for DM and HT. Peripheral blood analysis and patients’ characteristics are presented in Table 1.

**Table 1:** Demographic and peripheral blood analysis data of the patients.

Variables	Mean±SD
Age	66.6±8.7
Sex (n) (M/F)	62/12
Comorbidity (yes, no)	32/42
Forced expiratory Volume 1. second (%)	61.5±6.7
Body mass index (kg/m²)	24.3±5.4
Troponin-T	23±20
Pro-B-type natriuretic peptide	2023±4034
Vascular endothelial growth factor	91.05±70.70
Soluble urokinase plasminogen activator receptor	955.8±571.1

The mean LVEF was 62.3±5.8, and the mean TAPSE/sPAP was 0.48±0.15. RV characteristics are presented in Table 2.

**Table 2:** Right ventricular characteristics of the patients.

Right ventricular variables	Mean±SD
Right ventricular outflow tract diameter	31.5±4.2
Right ventricular end-diastolic diameter basal	38.2±5.5
Right ventricular end-diastolic diameter mid	28.3±6.2
Right ventricular end-diastolic diameter long axis	60.3±7.7
Right ventricular end-diastolic area	16.8±4.1
Right ventricular end-systolic area	9.7±3
Fractional area change	42.2±7.5
Right atrium area	14.1±4.5
Tricuspid regurgitation peak velocity	3.1±0.41
Tricuspid annular plane systolic excursion (TAPSE)	20.1±3.2
Systolic pulmonary artery pressure (sPAP)	45.8±12.7
TAPSE/sPAP	0.46±0.14
Tricuspid E	60.5±16
Tricuspid A	62.3±14.3
Tricuspid E/A	0.99±0.26
Deceleration time	121±51
RV systolic myocardial velocity of tricuspid annulus Sa	13.6±2.8
RV early diastolic velocity of tricuspid lateral annulus Ea	11.4±2.8
Late RV early diastolic velocity of tricuspid lateral annulus Aa	16.5±3.9
Right ventricular myocardial performance index	0.71±0.26

**Figure 1:**
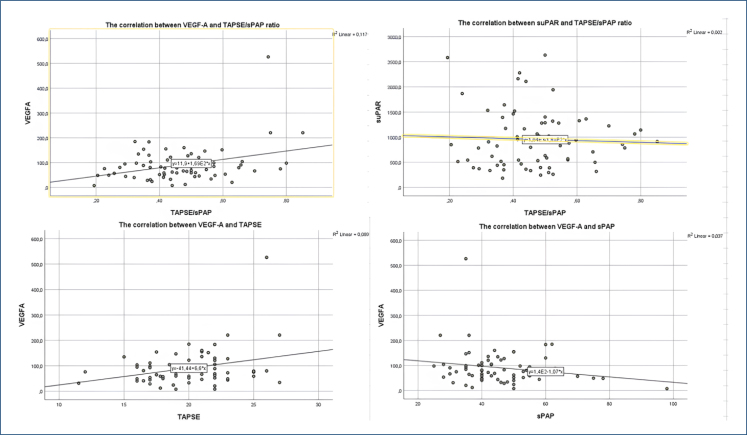
The correlation between vascular endothelial growth factor-A, soluble urokinase plasminogen activator receptor, and tricuspid annular plane systolic excursion/systolic pulmonary artery pressure.

Mean VEGF-A and suPAR were 91.05±70.7 and 955.8±571.1, and their Pearson correlation coefficients were 0.341 (p=0.004) and −0.045 (p=0.70), respectively. The linear regression model included four variables with significant correlation (VEGF-A, RAA, FAC, and RVOTD). Three steps were performed, and adjusted r^2^ was 0.22, 0.22, 0.20, and p<0.001 for each model step. VEGF-A and RVOTD remained in the last step (Durbin-Watson=1.82, collinearity tolerance=0.99, and VIF=1.004 for VEGF). It was detected a standardized coefficient beta of 0.322 (p=0.004) and a 95%CI 0.000–0.001 for VEGF-A (Table 3). When analyzing TAPSE and sPAP separately, there was a positive correlation between TAPSE and VEGF-A (p=0.01) and no correlation between sPAP and VEGF (p=0.45) (Figure 1).

**Table 3:** Coefficients of the linear regression model.

	US B	SE	S B	t	p	95%CI (lower, upper)		Tolerance	VIF
(Constant)	0.634	0.164		3.856	0.000	0.306	0.962		
RVOTD	-0.007	0.004	-0.221	-1.844	0.070	-0.016	0.001	0.782	1.279
RAA	-0.006	0.004	-0.177	-1.483	0.143	-0.013	0.002	0.789	1.267
FAC	0.002	0.002	0.121	1.080	0.284	-0.002	0.007	0.901	1.110
VEGFA	0.001	0.000	0.293	2.719	0.008	0.000	0.001	0.972	1.029
(Constant)	0.759	0.117		6.479	0.000	0.525	0.993		
RVOTD	-0.008	0.004	-0.244	-2.060	0.043	-0.016	0.000	0.806	1.241
RAA	-0.006	0.004	-0.193	-1.629	0.108	-0.014	0.001	0.802	1.247
VEGFA	0.001	0.000	0.309	2.895	0.005	0.000	0.001	0.991	1.009
(Constant)	0.759	0.119		6.403	0.000	0.523	0.996		
RVOTD	-0.011	0.004	-0.328	-3.046	0.003	-0.018	-0.004	0.996	1.004
VEGFA	0.001	0.000	0.322	2.987	0.004	0.000	0.001	0.996	1.004

RVOTD: right ventricular outflow tract; RAA: right atrium area; FAC: fractional area change; VEGFA: vascular endothelial growth factor A; US B: unstandardized B; VIF: variance inflation factor.

## DISCUSSION

In our study, an association between the TAPSE/sPAP ratio and VEGF-A was detected but not with suPAR. These results could be related to several reasons. suPAR is not a disease-specific diagnostic marker, considering its non-specific associations with immune activity and prognosis in miscellaneous diseases and conditions^11^. As for VEGF, it is released in hypoxic conditions^5^. There are several reasons to increase VEGF levels in COPD, such as hypoxemia, remodeling, and inflammation.

Nitric oxide (NO) and endothelin-1 are increased in response to hypoxemia^12^. This causes the contraction of smooth muscle cells and increases cell proliferation by inhibiting anti-mitogenic factors, NO and prostacyclin, and increasing the production of mitogenic stimuli such as VEGF^13^. However, this response varies from patient to patient. As a result of this variability, in COPD, even in severe disease, PH was seen in only 5% of the patients^1^.

A study performed in myocardial infarction (MI) detected that LVEF level was lower in the MI group; VEGF levels were significantly higher in this group compared to the control group (p<0.05). In addition, LVEF was negatively correlated with VEGF^14^. We compared VEGF levels and RH characteristics in our study. It was thought that similar aspects in these studies were most likely a response of VEGF increase to the secondary hypoxemic effect.

Pako et al. compared VEGF and RH parameters, including 20 patients with IPAH and 15 healthy participants. VEGF levels tended to be higher in patients with IPAH compared to the healthy group, and VEGF levels did not correlate with sPAP or others. Similar to our study, VEGF levels correlated to the TAPSE/sPAP ratio; however, when Pearson correlation analysis was performed separately, VEGF levels were associated with TAPSE and not with sPAP. Our study results were highly similar, but our study was carried out in group E COPD patients and used TTE, contrary to the above study, which was based on RHC and consisted of patients with IPAH^15^.

A study included 107 PH patients (48 with PAH, 5 with PH associated with left heart disease, 4 with PH associated with lung disease, and 50 with group 4 PH). Serum VEGF-A165b was significantly the highest in the lung disease PH (p<0.001)^16^. In the study, VEGF-A was detected to have increased plasma in lung disorders associated with PH. VEGF-A levels in our patients also increased and correlated with the TAPSE/sPAP ratio, which was recommended in the guideline concerning additional TTE signs suggestive of PH. Most probably, it was thought to contribute to the remodeling of lung tissue and smoking.

In a study by Mirna et al., 88 patients diagnosed with PH and 74 controls were included. suPAR levels were significantly different among the five groups of PH. However, the levels were primarily associated with group 2 PH. Moreover, a correlation between suPAR levels and echocardiographic signs such as RAA, TAPSE, and others was detected; however, they did not detect a correlation between suPAR and mPAP^10^. We could not show a correlation between suPAR and RH parameters. The above study did not explain the characteristics of the patients (22 patients and the remaining had other respiratory diseases). Our patients were in group E COPD, with both symptomatic conditions and a history of hospitalization. While the mean suPAR levels of our patients were 955 pg/mL, in the mentioned study, it was 4,878 pg/mL (median). However, the above study included some patients (nearly 50%) who had chronic kidney disease (CKD). Some studies showed that suPAR levels increased in CKDs^17^. suPAR levels may have increased due to comorbid conditions in the study. suPAR was associated with organ damage, remodeling, and inflammatory processes. In addition, there may also be other factors that affect the suPAR levels in the patients.

### Limitations

We performed only the echocardiographic characteristics of the patients and biomarkers; not evaluating them with RHC parameters is a limitation.

## CONCLUSION

We showed that plasma VEGF-A levels were correlated to TAPSE/sPAP. The positive correlation is important since the TAPSE/sPAP ratio is one of the additional echocardiographic signs suggestive of PH. The correlation was primarily related to TAPSE. We would expect a negative correlation between suPAR and the ratio, but there was no significant correlation.

## Data Availability

These are available from the corresponding author upon reasonable request.
